# Prognostic role of the neutrophil-to-lymphocyte ratio in pancreatic cancer: a meta-analysis

**DOI:** 10.1038/srep11026

**Published:** 2015-07-31

**Authors:** Hao Cheng, Feiwu Long, Mukesh Jaiswar, Lie Yang, Cun Wang, Zongguang Zhou

**Affiliations:** 1 Department of Gastrointestinal Surgery, West China Hospital, Sichuan University, Chengdu 610041, Sichuan Province, People’s Republic of China; 2 Institute of Digestive Surgery and State Key Laboratory of Biotherapy, West China Hospital, Sichuan University, Chengdu 610041, Sichuan, People’s Republic of China

## Abstract

The relationship between the neutrophil-to-lymphocyte ratio (NLR) and tumours as a prognostic factor has been reported in many studies. In this meta-analysis, we evaluated the prognostic role of the NLR in pancreatic cancer (PC). A systematic search was performed in PubMed and Embase for relevant studies. Data from and characteristics of each study were extracted. A meta-analysis was performed to analyse the prognostic role of the NLR using the hazard ratio (HR) and 95% confidence intervals (95% CI). As a result, a total of 2035 patients in 9 cohorts were included in this meta-analysis. The pooled HR of 1.587 (95% CI: 1.411–1.785, p < 0.01) showed that patients with an elevated NLR were expected to have shorter overall survival (OS) after treatment. This meta-analysis suggests that an elevated NLR can be used as a predictor of survival in patients with pancreatic cancer.

Pancreatic cancer (PC) results in approximately 227,000 deaths per year^1^ and ranks as the fifth most common cancer and the fourth leading cause of cancer-related mortality worldwide^2^. The prognosis of pancreatic cancer is poor, and the majority of patients survive less than 1 year after diagnosis. Virchow in 1863 first reported the association between inflammation and cancer^3^. Recent investigations revealed inflammatory markers as a predictor of prognosis of different types of cancers. Pretreatment serum-based inflammatory markers, such as NLR, platelet to lymphocyte ratio (PLR) and C-reactive protein (CRP), have been linked to the prognosis of different types of cancer^4^,^5^. As a marker of systemic inflammatory response, the NLR has been investigated as an effective prognostic indicator for several types of cancer. Some studies have revealed that an elevated NLR in patients with pancreatic cancer was linked to a poor prognosis, but results contradicting this connection have been presented by some studies, such as that by Hamed, Sanjay and Clark^6^,^7^,^8^. The purpose of this meta-analysis was to examine the link between the NLR and the prognosis in pancreatic cancer.

## Results

### Selection and characteristics of included studies

**Figure 1:**
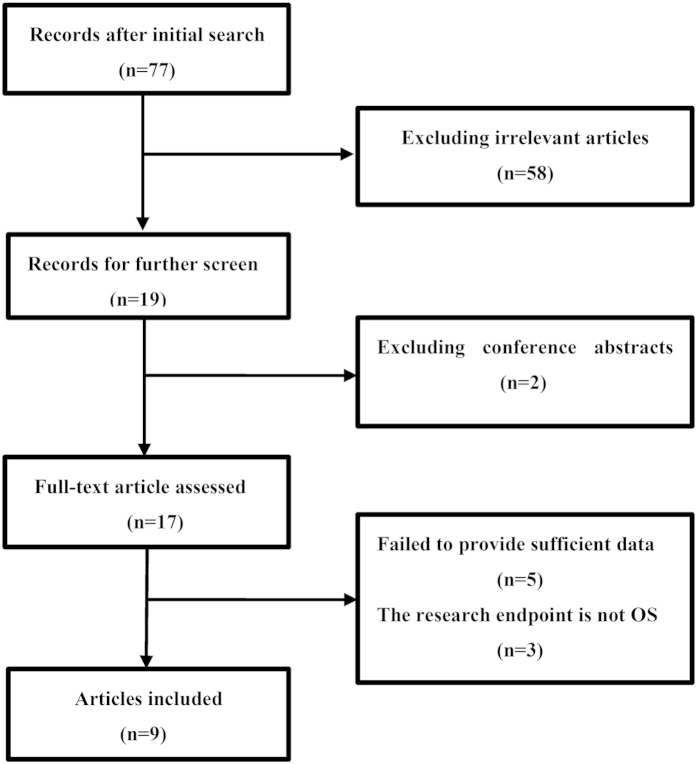
A flow chart outlining study selection.

A flow chart of the literature search is shown in Fig. 1. The initial search algorithm retrieved a total of 77 studies. After the first review, only 19 studies related to the NLR and the prognosis of PC were further evaluated. Of those studies, 10 reports were excluded for the following reasons: the endpoint of 3 studies was cancer-specific survival (CSS) or disease-free survival (DFS); 5 articles did not provide sufficient data for estimating the HR and 95% CI; and 2 articles were conference abstracts without detailed data. Thus, **9** studies^9^,^10^,^11^,^12^,^13^,^14^,^15^,^16^,^17^ published between 2007 and 2014 were included in our meta-analysis. The characteristics of the included studies are summarized in Table 1. A total of 2035 patients were included. The studies came from the UK (n = 1), China (n = 4), Japan (n = 2), Ireland (n = 1) and Australia (n = 1). The NLR was calculated on the basis of pretreatment laboratory data using the white blood cell (WBC) counts from the included studies. Eight of the 9 studies used multivariate analysis^13^.

### Meta-analysis results

**Figure 2:**
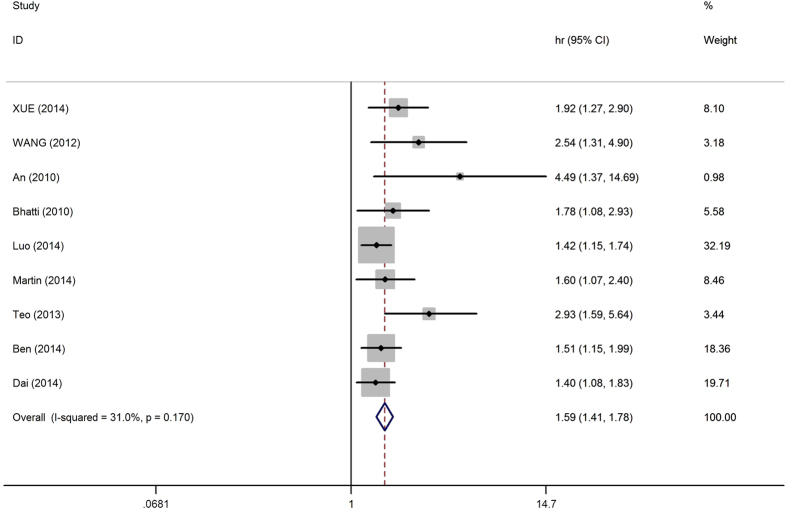
Forest plot of HR and 95%CI for each study. Fixed-effects pooled HR = 1.59, 95% CI = 1.41 − 1.78, P < 0.01; I^2^ = 31.0%, P_heterogeneity =_ 0.170.

Because the heterogeneity test showed that minor heterogeneity exists (I^2^ = 31.0%, p = 0.170) between the studies, a fixed-effects model was used for the analysis. A pooled HR of 1.587 (95%CI: 1.411–1.785, p < 0.01) showed that patients with an elevated NLR were expected to have shorter OS after treatment (Fig. 2). Because of relatively minor heterogeneity, we did not conduct further meta-regression or subgroup analysis.

### Publication bias

**Figure 3:**
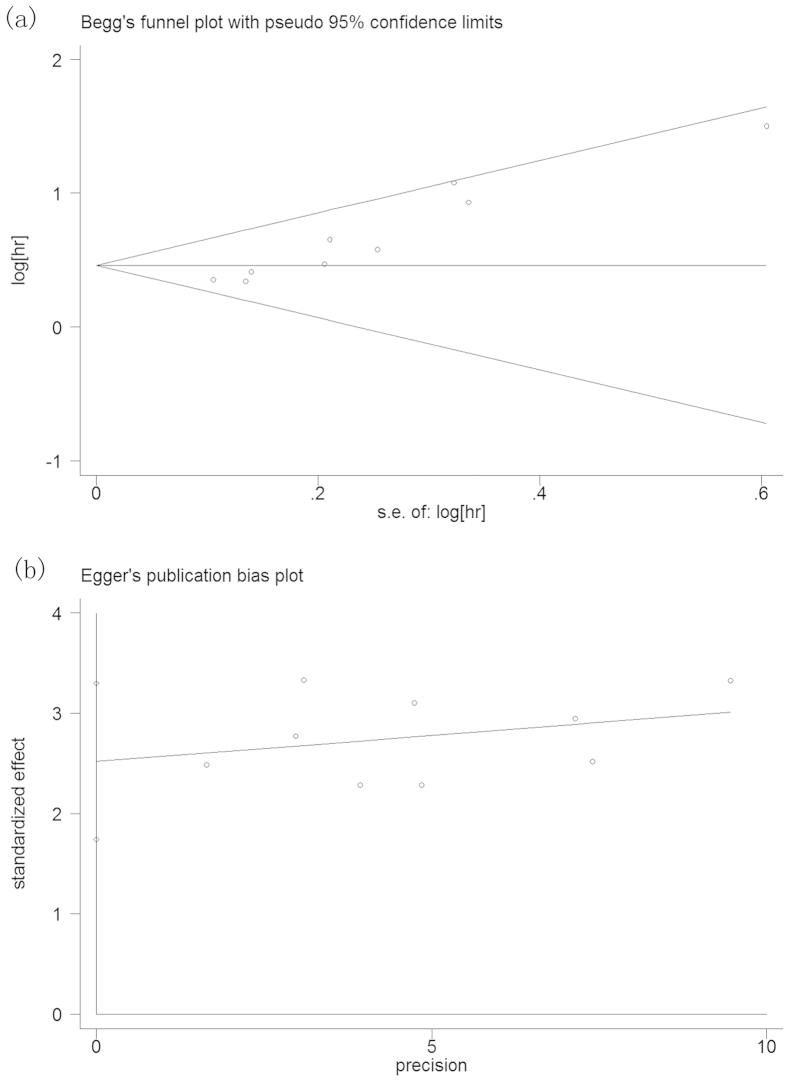
Begg’s (**a**) and Egger’s (**b**) funnel plot for the assessment of potential publication bias.

**Figure 4:**
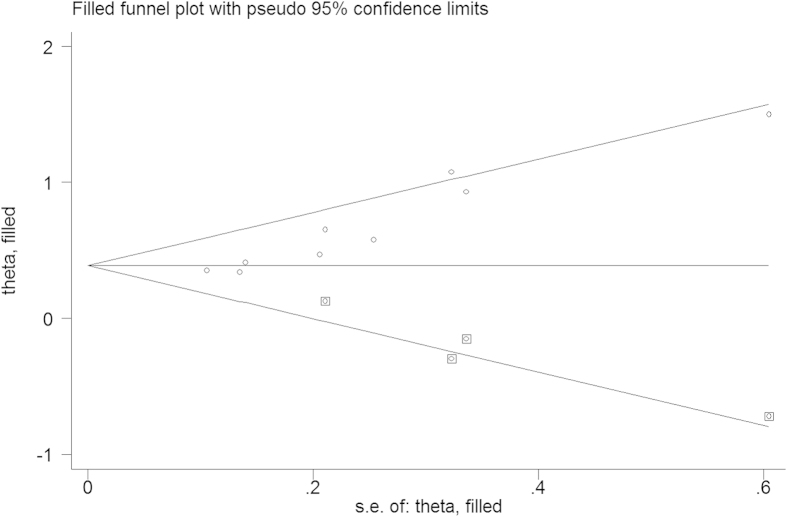
Trim-and-fill funnel plot for identifying the source of publication bias.

A publication bias estimate was used to evaluate the reliability of the meta-analysis results. A funnel plot (Fig. 3) was constructed, and the Begg’s test and Egger’s test showed that Pr > |z| = 0.002 and P > |t| = 0.000, respectively. The results revealed publication bias in this meta-analysis. To identify the source of publication bias, we used the trim-and-fill method (Fig. 4). As a result, there were four studies hypothetically remained unpublished, and the recalculated pooled HR of 1.475 (95%CI: 1.322-1.645, p < 0.01) indicated a positive outcome even though publication bias still exists.

## Discussion

The relationship between inflammation and tumours has been well established. Tumours not only can develop under the stimuli of inflammation, such as in HBV—induced hepatic cancer, but also can induce systemic and local inflammatory responses that may provide a favourable microenvironment for tumour invasion and metastasis^3^,^18^,^19^. Growing evidence has demonstrated that the association between inflammation and tumours could be applied to the prevention and treatment of cancer, such as anti-inflammation therapy for bladder cancer^20^,^21^. The NLR, which can be readily determined using a widely available peripheral blood test, is drawing increasing attention. An elevated NLR is usually caused by neutrophilia and lymphopenia. Lymphopenia indicates disease severity^22^,^23^ and is linked to the immune escape of tumour cells from tumour-infiltrating lymphocytes (TIL)^24^,^25^. It has been shown that elevated levels of tumour-infiltrating lymphocytes in the primary tumour site are associated with good prognosis^26^. It has also been reported that tumour cells can inhibit cytotoxic T lymphocyte infiltration by producing immunosuppressive cytokines, such as vascular endothelial growth factor (VEGF), transforming growth factor–β (TGF—β), or IL—10, and by reducing IL—2, a cytokine that can maintain cytotoxic T lymphocyte function^27^. Conversely, neutrophils have been reported to be the primary source of circulating VEGF, which has been shown to have a critical role in tumour-related angiogenesis and therefore has a close relationship with vascular invasion and metastasis in cancers^28^. Neutrophils from healthy donors were found to inhibit the cytolytic ability of lymphocytes when cocultured *in vitro*, and the magnitude of suppression was related to the number of neutrophils^29^. An elevated NLR generates a favourable immune microenvironment that promotes vascular invasion and suppression of the host immune system. Therefore, an elevated NLR is associated with poor prognoses.

To our knowledge, this meta-analysis, which included 9 cohorts and 2035 patients, is the first to identify the prognostic role of a pretreatment peripheral blood NLR in pancreatic cancer. According to the results, an elevated NLR predicts a poor prognosis and shorter OS in pancreatic cancer patients.

The meta-analysis had some limitations that call for cautious interpretation of the results. First, only 9 studies were included, and, because of insufficient data, studies^6^,^7^,^8^ with negative results were excluded. Second, the cut-off value for defining high NLR in each included study was not the same (Table 1), which may have contributed to heterogeneity. Third, some studies provided only a Kaplan-Meier curve and did not report HR or a 95% CI. Theoretically, these factors could be obtained through estimation from the curve, but that approach can be inaccurate. Therefore, we removed articles that did not meet our criteria from the analysis^6^,^30^,^31^. Fourth, besides articles with OS as an endpoint, we also searched for articles with CSS or DFS as an endpoint that focused on the relationship between NLR and PC prognosis, but we found only three articles^32^,^33^,^34^ (CSS: n = 2, DFS: n = 1). Given the small number, we did not include these articles in the meta-analysis.

In conclusion, this meta-analysis suggests that an elevated NLR can be used as a predictor of poor survival in patients with pancreatic cancer that is easily accessible and has a low cost. However, because of the study’s limitations, more multi-centre prospective cohorts need to be conducted to validate the role of the NLR in cancer.

## Methods

### Search strategy

We systematically searched PubMed and Embase with a search strategy based on the terms “NLR,” “neutrophil-to-lymphocyte ratio,” “neutrophil to lymphocyte ratio,” or “neutrophil lymphocyte ratio” and “pancreatic cancer,” “pancreatic carcinoma,” “pancreatic tumor,” or “pancreatic adenocarcinoma”. The last search was performed on January 16, 2015. To retrieve additional potentially eligible studies, the rences of articles and reviews were also examined.

### Inclusion and exclusion criteria

Studies fulfilling the following criteria were included in the meta-analysis: (1) the NLR was measured with pretreatment serum-based methods; (2) the association between NLR and OS in patients with PC was reported; (3) hazard ratio (HR) and 95% CI were reported; and (4) all included patients were pathologically diagnosed and did not have any tumours besides pancreatic tumours. The following publications were excluded: (1) studies that did not report HR or 95% CI; (2) letters, reviews, expert opinions, or case reports; and (3) studies that had a sample size less than 50.

### Data extraction

The following items were collected from each study: first author’s name, year of publication, country of the study population, sample size, sampling time, predominant treatment (surgical or nonsurgical), cut-off value for the NLR, PC stage and HRs with 95% CI.

### Statistical analysis

HRs with 95% CI from each study were extracted to generate a pooled HR. If both univariate and multivariate analyses were reported in the same study, multivariate analysis was chosen for the meta-analysis. We performed a test of heterogeneity using Cochran’s Q test and Higgins I-squared statistic. P ≥ 0.10 and I^2^ ≤ 50% were considered the values that indicated homogeneity, and a fixed-effects model was subsequently applied. P < 0.10 and I^2^ > 50% were considered the values that indicated severe heterogeneity, and the pooled HR was subsequently calculated by applying the random-effects model. Begg’s funnel plot and Egger’s linear regression test were used to evaluate publication bias. All statistical analyses were carried out using STATA 12.0 (STATA Corporation, College Station, TX, USA).

## Additional Information

**How to cite this article**: Cheng, H. *et al.* Prognostic role of the neutrophil-to-lymphocyte ratio in pancreatic cancer: a meta-analysis. *Sci. Rep.* **5**, 11026; doi: 10.1038/srep11026 (2015).

## Figures and Tables

**Table 1:** Characteristics of all identified studies.

Study	Year	Country	Treatment	HR(95%CI)	Multivariate analysis	Result	Endpoint
Xue^14^	2014	Japan	nonsurgery	1.92(1.27 – 2.9)	Yes	Positive	OS
Wang^11^	2012	China	Surgery & nonsurgery	2.537(1.313 – 4.902)	Yes	Positive	OS
Luo^10^	2014	China	nonsurgery	1.42(1.15 – 1.74)	Yes	Positive	OS
Martin^15^	2014	Australia	nonsurgery	1.60(1.07 – 2.40)	Yes	Positive	OS
An^12^	2010	China	surgery & nonsurgery	4.489(1.372 – 14.692)	Yes	Positive	OS
Bhatti^13^	2010	UK	surgery	1.78(1.09 – 2.93)	No	Positive	OS
Teo^9^	2013	Ireland	nonsurgery	2.93(1.59 – 5.64)	Yes	Positive	OS
Ben^17^	2014	China	Surgery	1.51(1.15 – 1.99)	Yes	Positive	OS
Dai^16^	2014	China	Surgery & nonsurgery	1.404(1.078 – 1.830)	Yes	Positive	OS

Study	sample	sample of elevated NLR	NLR cut-off	sample of distant metastasis	stage	sampling time
Xue^14^	252	36	4	83	Advanced	pretreatment
Wang^11^	177	12	3, 4	0	Ib-IV	pretreatment
Luo^10^	403	194	3.1	309	Advanced	pretreatment
Martin^15^	124	60	5	84	Advanced	pretreatment
An^12^	89	18	5	–	III- IV	pretreatment
Bhatti^13^	84	13	4	0	Early	pretreatment
Teo^9^	85	58	3	47	Advanced	pretreatment
Ben^17^	381	267	2	0	I-III	pretreatment
Dai^13^	440	300	2	143	I-IV	pretreatment

“-”: nor reported; “OS”: overall survival.
